# Impact of age at immigration on neuropsychological assessments among older South Asian Americans

**DOI:** 10.1002/alz.088313

**Published:** 2025-01-03

**Authors:** Karthik Kota, Jasdeep Hundal, Victor Sotelo, Allison Dawson, Shromona Sarkar, William T. Hu

**Affiliations:** ^1^ Rutgers‐Robert Wood Johnson Medical School, New Brunswick, NJ USA; ^2^ Jersey Shore University Medical Center, Neptune Township, NJ USA; ^3^ Rutgers Institute for Health, Health Care Policy, and Aging Research, New Brunswick, NJ USA; ^4^ Cancer Institute of New Jersey, New Brunswick, NJ USA

## Abstract

**Background:**

South Asian (SA) Americans are underrepresented in US Alzheimer’s disease and related dementias (ADRD) research. Despite sharing common languages, SA Americans differ from those in South Asia in education, health‐related behaviors, and environmental exposures. NYC/NJ‐area SA communities overwhelmingly revealed English‐based neuropsychological testing preference given that many speak non‐Hindi languages. Further complicating testing is evidence of non‐cognitive/linguistic factors influencing SA adults’ performance. For example, mean performance on the language‐independent Trail Making Test A (TMT‐A) among older adults across five Indian cities was <1^st^ percentile. We thus recruited an older SA American cohort without subjective cognitive complaints to undergo neuropsychological testing, correlating performance with immigration‐related variables.

**Method:**

65 SA participants completed neuropsychological testing, assessing memory (logical memory, CERAD word list, Benson complex figure), attention/speed (forward digit span [FD], TMT‐A), executive (reversed digit span [RD], TMT‐B, symbol digit substitution test [SDST], letter‐guided fluency [FAS]), language (Multilingual Naming Test [MINT], Boston Naming Test [BNT‐60], and visuospatial functions (Judgement of Line Orientation [JOLO]). Z‐scores derived from the National Alzheimer’s Coordinating Center calculators underwent linear regression modeling to determine influence from age (first learning English, at immigration), years in the US, and education (native country vs US).

**Result:**

Participants (30 women and 35 men) had a median age of 65 years (range 24‐86) and median education of 18 years (range 12‐20), with a median 16 in home country (range 3‐20); they were a median age of 30 (range 8‐69) years at immigration and had a median US residency of 32 years (range 4‐59). Age at immigration (story recall, TMT‐A, MINT, BNT, category fluency‐animals, JOLO) and years in the US (category fluency‐vegetables, word list learning and recall) had the greatest influence on neuropsychological performance, and two tests (story learning, letter‐guided fluency) were influenced by both. Five tests (Benson complex figure immediate and delayed recall, FD, RD, TMT B) were uninfluenced by immigration‐related factors.

**Conclusion:**

In addition to age, gender, and education, neuropsychological performance interpretation among SA Americans must account for age at immigration, years in the US, or both. Acculturation and familiarity with aptitude testing – potentially in language‐independent ways – likely influenced SA Americans’ neuropsychological analysis outcomes.

